# Optimizing Extraction Conditions of Free and Bound Phenolic Compounds from Rice By-Products and Their Antioxidant Effects

**DOI:** 10.3390/foods7060093

**Published:** 2018-06-13

**Authors:** Maria Irakli, Fotis Kleisiaris, Kalliopi Kadoglidou, Dimitrios Katsantonis

**Affiliations:** Hellenic Agricultural Organization “Demeter”, Institute of Plant Breeding & Genetic Resources, P.O. Box 60411, GR-57001 Thermi-Thessaloniki, Greece; cerealtech@cerealinstitute.gr (F.K.); kadoglidou@ipgrb.gr (K.K.); dikatsa@cerealinstitute.gr (D.K.)

**Keywords:** rice bran, rice husk, phenolics, antioxidant activity, response surface methodology, optimization extraction, free extraction, alkaline hydrolysis

## Abstract

Rice by-products are extensively abundant agricultural wastes from the rice industry. This study was designed to optimize experimental conditions for maximum recovery of free and bound phenolic compounds from rice by-products. Optimized conditions were determined using response surface methodology based on total phenolic content (TPC), ABTS radical scavenging activity and ferric reducing power (FRAP). A Box-Behnken design was used to investigate the effects of ethanol concentration, extraction time and temperature, and NaOH concentration, hydrolysis time and temperature for free and bound fractions, respectively. The optimal conditions for the free phenolics were 41–56%, 40 °C, 10 min, whereas for bound phenolics were 2.5–3.6 M, 80 °C, 120 min. Under these conditions free TPC, ABTS and FRAP values in the bran were approximately 2-times higher than in the husk. However, bound TPC and FRAP values in the husk were 1.9- and 1.2-times higher than those in the bran, respectively, while bran fraction observed the highest ABTS value. Ferulic acid was most evident in the bran, whereas *p*-coumaric acid was mostly found in the husk. Findings from this study demonstrates that rice by-products could be exploited as valuable sources of bioactive components that could be used as ingredients of functional food and nutraceuticals.

## 1. Introduction

Rice (*Oryza sativa* L.) is one of the most important crops worldwide and it is consumed as the main staple food for many countries, particularly in Asia. Among by-products of rice processing, are rice bran (RB) and husks (RH), which protect rice seeds during growth, accounting for 20% of the rough rice [1]. RB, the outer layer of brown rice accounting for 8–10% of its weight, serves as a good source of RB oil and bioactive compounds like γ-oryzanol, tocopherols, tocotrienols, phytosterols and phenolic compounds that have antioxidant activities, as well as other reported health-beneficial properties [2]. In recent years, several studies evaluate RB as a potential food ingredient designed to improve quality and nutrition profile [3,4].

RH consist mainly of cellulose, hemicellulose, lignin and hydrated silica. Traditionally it is used as a biofuel, incinerated in boilers to produce electricity, but it is mostly wasted because of its low-cost value as feedstock due to low digestibility, peculiarize, low bulk density, high ash/silica contents and its abrasive characteristics [5]. It is used mainly in various non-food applications, although it contains polyphenolic compounds to protect the inner materials from oxidative stress [6]. The phytochemical compounds and antioxidant activity of RB has been extensively reported [7,8,9]; however, RH is not deeply exploited as a source of phytochemicals. Although numerous studies have shown the potential applications of RB in functional foods development, limited information exists about the RH [10,11].

Many studies have focused on the determination of the soluble-free and in-soluble-bound phenolics of RB, but few studies are concentrated on the extraction optimization [9,12,13]. However, most of them are concentrated on free phenolics and no studies are available for the bound form. On the other hand, one empirical approach has been carried out in order to optimize phenolic compounds after acid/alkaline digestion of RH [10]. Up to now, no statistical methods exist on the optimization of ultrasonic-assisted extraction (UAE) conditions for both free and bound phenolic acids in rice by-products and this would be helpful for their exploitation in the food industry. Therefore, the aim of this study was to optimize UAE variables such as temperature, time and concentration of solvents or alkalines in order to maximize total phenolic content and antioxidant activity of free and bound phenolics from RB and RH by Response Surface Methodology (RSM). In addition, the phenolic acids composition in the extracts obtained at optimal conditions were analyzed by High Performance Liquid Chromatography (HPLC).

## 2. Materials and Methods

### 2.1. Chemicals

2,2′-azino-bis (3-ethylbenzthiazoline sulfonate) (ABTS), 2,4,6-tripyridyl-s-triazine (TPTZ), 6-hydroxy-2,5,7,8-tetramethylchroman-2-carboxylic acid (Trolox) and Folin–Ciocalteu reagent were purchased from Sigma–Aldrich (Steinheim, Germany). High purity analytical standards (>98%) of gallic acid (GA), vanillic acid (VA), syringic acid (SRA), 4-hydroxybenzoic acid (4HBA), *p*-coumaric acid (pCA), ferulic acid (FA), sinapic acid, protocatechuic acid, caffeic acid and chlorogenic acid were supplied by Sigma-Aldrich (Steinheim, Germany). All other reagents obtained from Chem-Lab (Zedelgem, Belgium) were of analytical or HPLC grade.

### 2.2. Plant Materials

Dried paddy rice grains from Axios variety were collected from the experimental farm of Institute of Plant Breeding and Genetic Resources (Thessaloniki, Greece). RH were obtained after dehulling rice grains using dehusker (Taka Yama, ΤW Grandeur Machinery Co., Taichung, Taiwan), whereas RB fraction including germ was produced after polishing brown rice using a debranning machine (Satake Engineering Co., Tokyo, Japan) and defatting on a Soxhlet device. Both fractions (hull and bran) were ground in a laboratory mill (ZM 1000, Retsch GmbH, Haan, Germany) to pass a 0.50 mm sieve and stored in a glass jar at −20 °C until analysis.

### 2.3. Ultrasound-Assisted Extraction

#### 2.3.1. Free Phenolic Extracts

200 mg powdered RB or RH was sonicated with 5 mL of different ethanol/water mixtures for different extraction times and temperatures in an ultrasonic bath (Elma, Fisher Scientific Ltd., Leicestershire, UK). Then, the extracts were centrifuged at 1500× *g* (10 min, 4 °C) and the supernatants were stored at −25 °C until analysis.

#### 2.3.2. Bound Phenolic Extracts

The extraction was performed by applying an alkaline hydrolysis according to Irakli et al. [14]. A sample of 200 mg of powdered RB or RH was extracted under the optimized conditions following the procedure described above. Supernatants were discarded and the remaining residue was used for the next alkaline hydrolysis step. The residue was suspended in 10 mL NaOH of different concentrations and the hydrolysis was performed under sonication at different times and temperatures. The mixture was then acidified to pH 2 with concentrated HCl, the supernatant was recovered by centrifugation at 1500× *g* for 5 min and then extracted three times with 25 mL ethyl acetate. The ethyl acetate fractions were mixed and vacuum evaporated at 40 °C and the residue was reconstituted in 5 mL of the ethanol/water mixture (50:50, *v*/*v*) and stored at −25 °C until analysis.

### 2.4. Total Phenolic Content Determination

The analyses of total phenolic contents (TPC) from free and bound fraction were performed using the Folin–Ciocalteu’s method according to Singleton et al. [15] with some modifications. Briefly, 0.2 mL of extract was transferred into a test tube and mixed with 0.8 mL of the Folin–Ciocalteu reagent. Ethanol/water mixture (50:50, *v*/*v*) was used as blank. After incubation for 2 min, 2 mL sodium carbonate (7.5% *w*/*v*) solution was added to the reaction mixture and the volume was adjusted to 10 mL with distilled water. The mixture was allowed to stand for 60 min in a dark place and then the absorbance at 725 nm was recorded. The analyses were performed in triplicate and results were expressed as mg of GA equivalents (GAE) per 100 g of sample.

### 2.5. ABTS Assay

Radical scavenging activity of rice by-products extracts against ABTS radical cation was evaluated according to the protocol of Re et al. [16] and appropriately adjusted. Briefly, ABTS^●+^ solution was obtained by reacting 2 mmol/L ABTS stock solution with 0.73 mmol/L potassium persulfate and the mixture was left to stand in the dark at room temperature for 12–16 h before use. The ABTS^●+^ solution was diluted with water to an absorbance at 734 nm of 0.70 ± 0.02. After the addition of 100 μL of phenolic extract to 3.9 mL of diluted ABTS^●+^ solution, the absorbance was measured against a blank at 734 nm after 4 min. Inhibition of ABTS radical cation (%) was calculated by using the following equation: Inhibition (%) = ((A_0_ − A_s_)/A_0_) × 100, where A_0_ is the absorbance of the blank sample and A_s_ is the absorbance of the sample at 4 min. The results were expressed as mg Trolox equivalents (TE) per 100 g of sample.

### 2.6. Ferric Reducing Antioxidant Power

The reducing power of extracts was determined according to the method of Benzie and Strain [17]. 0.1 mL aliquot of extract was reacted with 3 mL of FRAP solution at 37 °C for exactly 4 min under dark conditions. Readings of the colored product were then taken at 593 nm against blank and the results were expressed as mg TE per 100 g of sample.

### 2.7. HPLC Analysis of Phenolic Acids

Phenolic extracts from both free and bound fractions were filtered (pore size 0.2 μm) and the HPLC analysis was performed and applied to chromatographic conditions as described by Irakli et al. [14]. Detected phenolic acids in sample extracts were identified by comparison of their retention times with those of pure standards, and quantification was performed using the corresponding calibration curves. The results were expressed as mg per 100 g of sample.

### 2.8. Experimental Design

RSM using Box-Behnken Design (BBD) with three factors (*X*_1_, *X*_2_ and *X*_3_) on three levels (−1, 0, +1) was applied to optimize the conditions for extraction free and bound phenolics from RB and RH under the variables studied. The investigated independent variables for free phenolic extraction were ethanol concentration in water (*X*_1_ = 40–80%), extraction time (*X*_2_ = 10–60 min) and extraction temperature (*X*_3_ = 40–80 °C), whereas for bound phenolic extraction were NaOH concentration (X_1_ = 1–4 M), time of alkaline hydrolysis (*X*_2_ = 60–120 min) and temperature of hydrolysis (*X_3_* = 40–80 °C). The response values were TPC, ABTS and FRAP values. A total of 15 runs were performed with 3 center points. All extractions were performed in triplicate and the average values were taken as the response, *Y*. Experimental data were fitted to a second-order polynomial model and regression coefficients were obtained. A mathematical regression model for the response of *Y* (the predicted response) was fitted as follows:

$$Y=\beta_{0}+\overset{k}{\underset{i=1}{\sum }}\beta_{i}X_{i}+\overset{k}{\underset{i=1}{\sum }}\overset{k-1}{\underset{j=2}{\sum }}\beta_{ij}X_{i}X_{j}+\overset{k}{\underset{i=1}{\sum }}\beta_{ii}X_{i}^{2}$$

where *Y* is the measured response, *X*_i_ and *X*_j_ are the independent variables affecting the response and β_0_, β_i_, β_ii_, and *β*_ij_ are the regression coefficients for intercept, linear, quadratic and interaction terms, respectively.

An analysis of variance (ANOVA) with 95% confidence level was generated and the effect and regression coefficients of individual linear, quadratic and interaction terms were determined. The significances of all terms were judged statistically by computing the *F*-value at a probability *p* ≤ 0.05. The *p*-value of lack-of-fit was used to verify the adequacy of the model. The maximum value of each variable was set as a goal of the experiment. Statistical analysis was performed using the Minitab 17 (Minitab Inc., State College, PA, USA) software and fitted to a second-order polynominal regression model containing the coefficient of linear, quadratic and interaction terms.

## 3. Results and Discussion

### 3.1. Impact of Free Phenolics Extraction Parameters from Rice By-Products

In this study ethanol, methanol, acetone, hexane and ethyl acetate were tested as extraction solvents. Among them, ethanol and methanol had higher free phenolic contents than others [13]. However, ethanol was selected as an extraction solvent, as it is a green solvent, low-cost, completely biodegradable and easily available in high purity [18].

The experimental values of the responses of each experiment were listed in Table 1. According to the applied experimental design, TPC of the extracts obtained from RB and RH with UAE ranged from 206 to 464 mg GAE/100 g and 67 to 233, respectively. ABTS values ranged from 508 to 686 mg TE/100 g and 85 to 273 mg TE/100 g, whereas FRAP values ranged from 305 to 653 and 110 to 351 mg TE/100 g for RB and RH, respectively. According to these experimental ranges, maximum TPC and FRAP values were obtained at run 12 (80 °C, 60 min and 60% ethanol) and maximum ABTS values were observed in the run 10 (40 °C, 60 min and 60% ethanol). While the lowest TPC, ABTS and FRAP values were observed in the run 4 (60 °C, 60 min and 80% ethanol). On the other hand, the maximum TPC, FRAP and ABTS values in RH were obtained at run 7 (80 °C, 35 min and 40% ethanol) and run 3 (60 °C, 60 min and 40% ethanol), respectively, whereas the lowest one at run 4 (60 °C, 60 min and 80% ethanol).

It can be seen from the Table 2 that the variables with the highest effect in the free phenolic extracts of RB and RH were the linear terms of ethanol concentration (*X*_1_) and its quadratic term (*X*_1_^2^), while extraction time and temperature had a insignificant effect (*p* > 0.05). The model shows high significant and good fit for the most examined variables except TPC, presenting less variation around the mean (*R*^2^ ranged from 0.8001 to 0.9191). The non-significant lack-of-fit indicates that the model is adequate for the observed data at 95% confidence level.

**Figure 1:**
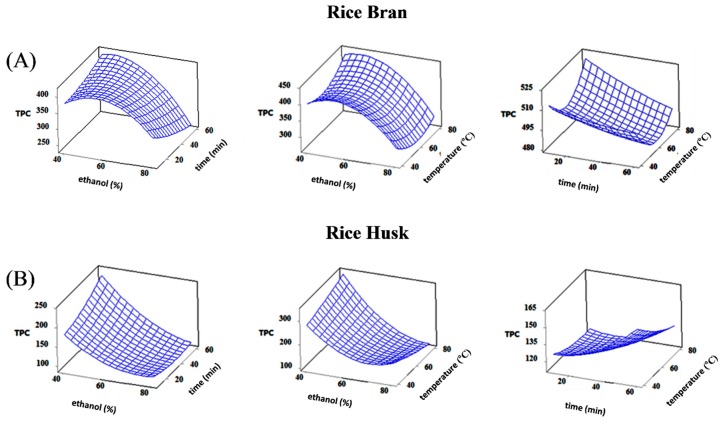
Effect of ethanol concentration, time and temperature extraction on total phenolic content (TPC, mg GAE/100 g) of free phenolics from (**A**) rice bran and (**B**) rice husk fractions.

The 3D response surface plots for TPC (Figure 1), ABTS radical scavenging activity and ferric reducing antioxidant power (Figures S1 and S2) demonstrate the relationship between extraction or hydrolysis parameters and responses. From the Figure 1, it can be seen that TPC values in RB extracts increased with the increase of the ethanol concentration and between 55–58% reached a peak. After that value, ethanol concentration had a negative effect on responses and this effect may be attributed to the change of solvent polarity. Longer extraction time at higher temperatures had insignificant effects (*p* > 0.05) on the TPC. According to these findings, we kept them at the minimum values (10 min and 40 °C) in order to shorten the time analysis and decrease the cost of energy. Similarly, Tabaraki and Nateghi [13] found that 65–67% ethanol concentration and its quadratic effect were the most pronounced factors influenced the TPC and antioxidant activity of RB under UAE.

On the other hand, the TPC and ABTS values in RH extracts decreased sharply as the ethanol concentration increased (Figure 1 and Figure S1). However, FRAP values increased with increased ethanol concentration up to 50%, followed by a sharp decrease afterwards (Figure S1). Additionally, time extraction of RH increased insignificantly (*p* > 0.05) with the increase of temperature up to 60 min for all responses. A similar trend was observed for the temperature extraction except for the TPC where a slight decrease was noticed as the temperature increased up to 80 °C. Similar to our results, Vadivel and Brindha [11] found that the best extraction time for UAE of phenolics from RH with 75% ethanol was 25 min.

### 3.2. Impact of Bound Phenolics Extraction Parameters from Rice By-Products

Significant amounts of phenolic compounds in foods are bound to cell walls via linking with cellulose, hemicellulose, lignin, pectin and rod-shaped structural proteins and acid/alkali hydrolysis is the most common means of releasing phenolic compounds by breaking ether and ester bonds [19]. Phenolic compounds are better released with alkaline hydrolysis than in acid hydrolysis conditions giving maximum phenolic recovery [11]. However, severe or prolonged alkaline treatment may result in degradation of the released phenolic acids, and different compounds may have variable susceptibility to degradation. In the current study, the effects of alkaline concentration (*X*_1_), hydrolysis time (*X*_2_) and temperature (*X*_3_) on the release of bound phenolic compounds from rice by-products were investigated (Table 1).

TPC of RB and RH after alkaline hydrolysis ranged from 185 to 340 mg GAE/100 g and 346 to 660 mg GAE/100 g, respectively. ABTS values ranged from 764 to 851 mg TE/100 g and 443 to 738 mg TE/100 g, whereas FRAP values ranged from 415 to 570 mg TE/100 g and 393 to 688 mg TE/100 g, respectively. According to these experimental ranges, the maximum responses were obtained at run 12 (80 °C, 120 min and 2.5 M), while the lowest were observed at run 1 (60 °C, 60 min and 1 M). Similarly, the maximum responses in RH extracts were obtained at run 12 (80 °C, 120 min and 2.5 M), however, the lowest values at run 5 (40 °C, 90 min and 1 M).

The ANOVA indicated that all the evaluated factors had a significant linear effect (*p* < 0.05) on the TPC, ABTS and FRAP values (Table 2). The model was insignificant (*p* > 0.05) and had good fit for all variables presenting a higher coefficient of determination (*R*^2^ ranged from 0.8915 to 0.9884) than the obtained results for the free phenolic extraction.

**Figure 2:**
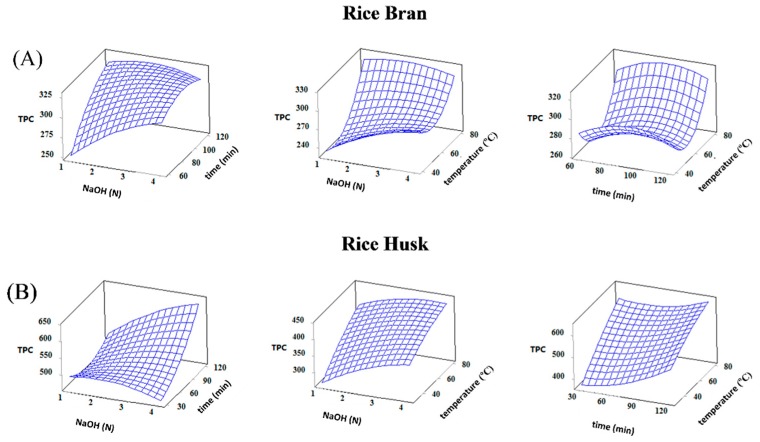
Effect of NaOH concentration, time and temperature hydrolysis on total phenolic content (TPC, mg GAE/100 g) of bound phenolics from (**A**) rice bran and (**B**) rice husk fractions.

The 3D response surface plots were constructed to illustrate the effects of independent variables and their interactions on the responses (Figure 2 and Figure S2). According to Figure 2, the TPC of RB extracts increased significantly with the increasing of NaOH concentration and hydrolysis time at 60 °C, but their interaction was insignificant. The same behavior was observed for the relationships of NaOH concentration and hydrolysis time at a constant time of 90 min as well as of time and temperature hydrolysis at a constant NaOH concentration of 2.5 M. However, the effect of NaOH concentration in the case of RH extract followed a different trend. The TPC increased with the increasing NaOH concentration up to 2.5 M and was followed by a decrease afterwards. These results have demonstrated that the alkaline concentration was a key factor in the release of the phenolic compounds from the cell walls of RH. This leads to the notifications that milder conditions may successfully lead to release of ester-linked phenolic acids, whereas more severe conditions can stimulate product degradation, likely due to redox reactions [20].

The effect of hydrolysis time on TPC from both substrates showed a similar trend except some little discrepancies. In the case of RB extracts, the TPC increased rapidly with an increase in the hydrolysis time up to 100 min and then it was constant until 120 min, whereas longer hydrolysis times are required in the RH. In agreement to our work, some previous research reports indicated that up to 120 min is appropriate for recovery polyphenols from different plant materials [11]. In addition, elevated hydrolysis temperatures favor the release of more phenolic compounds from both substrates, as can be seen in Figure 2. Hayat et al. [21] showed that the bound phenolic acids, as extracted by microwave-assisted extraction or UAE, gave better recoveries compared to conventional extractions. Therefore, higher amounts of phenolic compounds were extracted when hydrolysis was performed at a middle level of alkaline concentration for prolonged time and elevated temperatures.

### 3.3. Optimization of the Extraction and Hydrolysis Processes

In order to validate the adequacy of the model equation, a verification experiment was carried out under the optimized conditions for each substrate as well as for free and bound phenolics (Table 3). Under the optimal conditions for the RB, namely 40 °C, 10 min and 56%, resulted optimal free TPC (378 ± 5 mg GAE/100 g), ABTS (612 ± 24 mg TE/100 g) and FRAP values (606 ± 31 mg TE/100 g). These results are in the range of 2.51–3.59 mg GAE/g reported by Iqbal et al. [22] for commercially varieties of RB extracts, but lower than those reported by Tabaraki and Nateghi [13]. On the other hand, extraction at 40 °C for 60 min with 41% ethanol, can result in optimal free TPC (168 ± 3 mg GAE/100 g), ABTS (236 ± 11 mg TE/100 g) and FRAP values (338 ± 10 mg TE/100 g) from RH. Jha et al. [23] also reported similar values of TPC for extraction polyphenols from black RH.

Bound phenolics were performed under the optimal conditions for the RB (hydrolysis with 3.4 M NaOH at 80 °C for 110 min), resulted optimal TPC (366 ± 18 mg GAE/100 g), ABTS (818 ± 14 mg TE/100 g) and FRAP values (592 ± 19 mg TE/100 g). These results are in the range of 147.7–1914.2 mg GAE/100 g reported by Begum et al. [24] for the phenolic profile in bran of most cultivated rice varieties in India. In our study, the contribution of bound phenolics to the TPC was approximately 50% as the free fraction, as was already reported by several studies [8,25]. However, many studies reported a higher amount of bound phenolics than free phenolics et al. [25].

On the other hand, hydrolysis with 2.5 M NaOH at 80 °C for 120 min, can result in optimal bound TPC (696 ± 1 mg GAE/100 g), bound ABTS (723 ± 11 mg TE/100 g) and bound FRAP values (687 ± 16 mg TE/100 g) from RH. In contrast to RB, bound phenolic content in RH was approximately 4-times higher than free phenolics. Similarly, Butsat and Siriamornpun [26] showed that the phenolic content in RH °C occurred mostly in the bound form rather than free form.

The predicted results, obtained based on the model equation taking into account only the significant factors, matched well with the experimental results obtained using optimum extraction conditions with a good correlation. The difference between the values obtained by the model and experimentally was lower than 7%. These results confirm the predictability of the model for the extraction of free phenolics from RB and RH.

### 3.4. Comparison between RB and RH Phenolic Extracts

**Figure 3:**
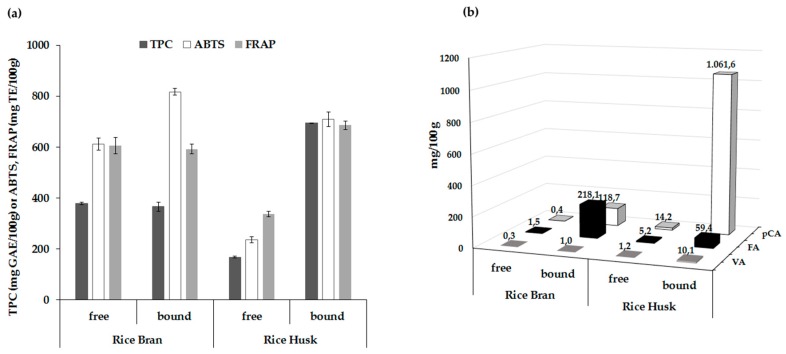
(**a**) Comparison of total phenolic content (TPC), ABTS radical scavenging activity and ferric reducing antioxidant power (FRAP) values of free and bound fractions of rice bran and husks. (**b**) Main phenolic acid composition of free and bound fractions of rice bran and husks.

TPC and antioxidant activity of free and bound extracts of rice by-products by applying the optimized conditions, are shown in Figure 3a. The bound TPC of RH fraction had the highest value (864 mg GAE/100 g) whilst its free fraction had the lowest TPC (168 mg GAE/100 g). Both RB fractions presented moderate TPC values with non-significant differences between free and bound extracts. Comparing the TPC of the RB and RH fractions, the total values (free and bound) of RH were 1.2-times higher than those of RB, indicating that RH would be an equally source of phenolics as RB.

Both bound fractions exhibited remarkably higher ABTS values when compared to free fractions, possibly due to high concentration of phenolic compounds released by alkali treatment. However, the bound extracts of RB fraction showed greater ABTS values (818 mg TE/100 g) than that of RH (710 mg TE/100 g), in contrast to the TPC. In free fractions, RB had relatively higher ABTS values (612 mg TE/100 g) than RH (236 mg TE/100 g), that was in accordance with Butsat and Siriamornpun [26]. There are no available data in the literature examining bound antioxidant activity in RB and RH in order to compare our findings. It is well known that other bioactive compounds such as tocopherols and γ-oryzanol present in RB contribute to antioxidant activity in contrast to the major antioxidant components found in RH fraction which may be phenolic acids [26,27]. The FRAP value of free extracts indicated that the RB fraction had the greatest reducing power (607 mg TE/100 g) followed by RH (338 mg TE/100 g), however the bound extracts of RH fraction gave higher reducing power (687 mg TE/100 g) than RB (592 mg TE/100 g). The results showed that the FRAP values were dependent on the variations in TPC.

### 3.5. HPLC Analysis of Phenolic Acids in Rice By-Products Extracts

The distribution of main phenolic acids namely VA, SRA, *p*CA and FA, found in the free and bound extracts of RB and RH, are given in Figure 3b. Minor phenolic acids were protocatechuic, caffeic and sinapic acids, whereas gallic and chlorogenic acids were not detected. The major phenolic acid in RB extracts is FA, whereas *p*CA followed by FA and SRA were the most abundant phenolic acids in RH. In the bound form of RB extract, FA was the most abundant phenolic acid followed by *p*CA, accounting for 64% and 35% of the total phenolic acids, respectively. On the other hand, the main bound phenolic acids in RH extracts were *p*CA, followed by FA and VA, accounting for 93%, 5% and 1% of the phenolic acids, respectively. Sun et al. [28] indicated that *p*CA is linked to lignin mainly by ester bonds, whereas FA is linked to lignin by both ether and ester bond. Hence, it is very likely that some phenolic esters are liberated during the saponification in alkaline solution. The results showed that bound FA in RB and RH extracts was on an average 10- and 150-times higher than free FA, respectively, while *p*CA in bound form was 75- and 300-times higher than free form, respectively. Nenadis et al. [10] also confirmed that *p*CA and FA were the major phenolic acids in from some rice varieties cultivated in Greece. As would be expected, RH contained substantial contents of phenolic acids, with higher bound phenolic acids than free phenolic acids.

## 4. Conclusions

In summary, the application of the optimized conditions to RB and RH showed that significant amounts of TPC could be recovered, leading to extracts with high ABTS scavenging efficiency and reducing power. The total antioxidant activity of RB was found to be higher than that of RH, although RB contained lower TPC than RH. This may be due to the fact that other bioactive compounds present in RB contribute to antioxidant activity in contrast to that of the major antioxidant components found in RH which may be phenolic acids. The overall results of this research demonstrated the potential of rice by-products to be an abundant source of natural antioxidants suitable for further development into dietary supplements and various food additives, leading to the development of innovative products of high economic importance.

## Figures and Tables

**Table 1:** Box-Behnken design for the independent variables and response values for free and bound phenolics.

Run	Independent Variables			Response Variables					
	*X*_1_	*X*_2_	*X*_3_	Rice Bran			Rice Husk		
	(% or M)	(min)	(^o^C)	TPC	ABTS	FRAP	TPC	ABTS	FRAP
1	40	10	60	409 ± 28	684 ± 12	537 ± 21	190 ± 8	227 ± 4	245 ± 2
	**1**	**60**	**60**	**185 ± 18**	**764 ± 24**	**415 ± 14**	**496 ± 14**	**543 ± 22**	**493 ± 10**
2	80	10	60	326 ± 25	614 ± 5	404 ± 22	142 ± 12	147 ± 9	152 ± 25
	**4**	**60**	**60**	**263 ± 15**	**810 ± 25**	**493 ± 23**	**447 ± 23**	**556 ± 7**	**506 ± 5**
3	40	60	60	401 ± 2	669 ± 12	595 ± 38	200 ± 5	273 ± 18	309 ± 7
	**1**	**120**	**60**	**245 ± 12**	**793 ± 28**	**475 ± 18**	**520 ± 17**	**580 ± 12**	**531 ± 10**
4	80	60	60	206 ± 12	508 ± 8	305 ± 19	87 ± 6	85 ± 8	110 ± 10
	**4**	**120**	**60**	**260 ± 23**	**825 ± 18**	**560 ± 32**	**631 ± 25**	**680 ± 40**	**630 ± 22**
5	40	35	40	412 ± 15	681 ± 5	475 ± 24	195 ± 5	261 ± 27	242 ± 4
	**1**	**90**	**40**	**216 ± 15**	**800 ± 35**	**446 ± 20**	**346 ± 16**	**443 ± 6**	**393 ± 3**
6	80	35	40	334 ± 6	598 ± 6	389 ± 14	103 ± 4	123 ± 28	140 ± 15
	**4**	**90**	**40**	**314 ± 17**	**825 ± 32**	**544 ± 12**	**465 ± 15**	**510 ± 20**	**460 ± 13**
7	40	35	80	407 ± 12	659 ± 14	586 ± 20	233 ± 10	235 ± 9	351 ± 11
	**1**	**90**	**80**	**282 ± 12**	**817 ± 30**	**512 ± 29**	**522 ± 12**	**635 ± 8**	**585 ± 19**
8	80	35	80	277 ± 8	558 ± 12	404 ± 21	67 ± 2	143 ± 3	136 ± 1
	**4**	**90**	**80**	**321 ± 22**	**833 ± 18**	**551 ± 21**	**569 ± 12**	**732 ± 18**	**682 ± 7**
9	60	10	40	380 ± 5	613 ± 6	430 ± 25	112 ± 5	145 ± 11	163 ± 9
	**2.5**	**60**	**40**	**220 ± 14**	**788 ± 28**	**450 ± 28**	**349 ± 10**	**468 ± 22**	**418 ± 13**
10	60	60	40	411 ± 16	686 ± 8	545 ± 36	193 ± 3	230 ± 10	301 ± 2
	**2.5**	**120**	**40**	**265 ± 25**	**804 ± 35**	**495 ± 24**	**476 ± 16**	**523 ± 5**	**473 ± 16**
11	60	10	80	434 ± 22	679 ± 10	565 ± 32	83 ± 5	201 ± 25	249 ± 24
	**2.5**	**60**	**80**	**295 ± 24**	**820 ± 25**	**525 ± 32**	**620 ± 10**	**663 ± 45**	**649 ± 10**
12	60	60	80	464 ± 12	628 ± 18	653 ± 40	144 ± 8	240 ± 15	350 ± 20
	**2.5**	**120**	**80**	**340 ± 20**	**851 ± 30**	**570 ± 20**	**660 ± 18**	**738 ± 18**	**688 ± 3**
13	60	35	60	395 ± 8	654 ± 9	520 ± 10	110 ± 2	233 ± 20	315 ± 22
	**2.5**	**90**	**60**	**278 ± 16**	**800 ± 28**	**508 ± 28**	**533 ± 13**	**616 ± 8**	**566 ± 11**
14	60	35	60	354 ± 10	648 ± 24	487 ± 8	145 ± 7	210 ± 16	260 ± 10
	**2.5**	**90**	**60**	**260 ± 14**	**797 ± 14**	**490 ± 12**	**544 ± 18**	**615 ± 18**	**565 ± 14**
15	60	35	60	403 ± 20	674 ± 10	565 ± 28	109 ± 2	224 ± 17	305 ± 3
	**25**	**90**	**60**	**239 ± 16**	**810 ± 32**	**469 ± 30**	**462 ± 15**	**580 ± 22**	**471 ± 16**

For phenolic extraction: *X*_1_, ethanol concentration; *X*_2_, extraction time; *X*_3_, temperature extraction. For phenolic hydrolysis: *X*_1_, NaOH concentration; *X*_2_, hydrolysis time; *X*_3_, temperature hydrolysis. TPC: total phenolic content (mg GAE/100 g); ABTS: ABTS radical scavenging activity (mg TE/100 g); FRAP: ferric reducing antioxidant power (mg TE/100 g). Bold values indicate bound phenolics.

**Table 2:** *F*-values and coefficient of determination of the predicted second order polynomial models for free and bound phenolics.

Source	Rice Bran						Rice Husk					
	TPC		ABTS		FRAP		TPC		ABTS		FRAP	
	Free	Bound	Free	Bound	Free	Bound	Free	Bound	Free	Bound	Free	Bound
Model	3.12	6.75 *	4.86 *	4.53 *	4.06 *	8.39 *	2.22	5.65 *	6.31 *	47.39 ***	5.87 *	9.15 *
*X*_1_	16.05 **	17.69 **	28.34 **	13.50 *	19.59 **	31.85 **	15.21 **	2.73 *	44.03 ***	37.72 **	27.68 **	6.92 *
*X*_2_	0.39	7.37 *	1.62	7.57 *	1.10	16.89 **	0.82	8.79 *	2.10 *	41.71 ***	5.46	5.87 *
*X*_3_	0.09	16.71 **	0.47	10.22 *	5.60	17.71 **	0.50	32.16 **	0.63	334.03 ***	4.65	66.80 ***
*X*_1_^2^	6.71 *	0.76	5.58	0.02	6.64 *	0.00	1.62	0.58	3.66	3.64	10.87 *	0.29
*X*_2_^2^	0.18	1.40	0.18	0.36	0.14	0.10	0.19	1.45	0.88	0.07	0.81	0.73
*X*_3_^2^	1.94	10.80 *	0.01	8.09 *	0.23	8.20*	0.03	0.40	0.16	0.91	0.07	0.11
*X*_1_ *X*_2_	1.68	2.65	2.76	0.34	2.01	0.03	0.58	3.25	4.24	7.29	1.69	1.34
*X*_1_ *X*_3_	0.37	2.36	0.11	0.15	0.76	2.53	0.73	0.33	0.74	0.85	1.92	0.16
*X*_2_ *X*_3_	0.01	0.00	4.99	0.43	0.06	0.00	0.95	0.95	0.74	0.38	0.10	0.05
Lack-of-fit	3.74	0.96	6.37	4.09	2.61	0.87	4.89	1.00	8.15	0.36	2.66	0.12
*R* ^2^	0.8490	0.8949	0.8979	0.8915	0.8980	0.9379	0.8001	0.9104	0.9191	0.9884	0.9135	0.9428

*** Significant at *p* ≤ 0.001; ** significant at *p* ≤ 0.01; * significant at *p* ≤ 0.05; *X*_1_: ethanol or NaOH concentration; *X*_2_: extraction or hydrolysis time: *X*_3_: temperature extraction; TPC: total phenolic content (mg GAE/100 g); ABTS: ABTS radical scavenging activity (mg TE/100 g); FRAP: ferric reducing antioxidant power (mg TE/100 g).

**Table 3:** Experiment data of the validation of predicted value at estimated optimum conditions.

	Rice Bran				Rice Husk			
	Free Phenolics		Bound Phenolics		Free Phenolics		Bound Phenolics	
Response variable	Predicted Value ^1^	Experimental Value ^2^	Predicted Value ^1^	Experimental Value ^2^	Predicted Value ^1^	Experimental Value ^2^	Predicted Value ^1^	Experimental Value ^2^
TPC	417	378 ± 15	339	366 ± 18	191	168 ± 13	647	696 ± 1
ABTS	647	612 ± 24	838	818 ± 14	262	236 ± 11	742	723 ± 11
FRAP	553	606 ± 31	569	592 ± 19	324	338 ± 10	680	687 ± 16

^1^ Calculated using the predicted equations for response variables at optimal condition, ^2^ Means of three replicates ± standard deviation; TPC: total phenolic content (mg GAE/100 g); ABTS: ABTS radical scavenging activity (mg TE/100 g); FRAP: ferric reducing antioxidant power (mg TE/100 g).

## Supplementary Materials

Supplementary materials are available at http://www.mdpi.com/2304-8158/7/6/93/s1. Figure S1. Effect of ethanol concentration, time and temperature extraction on ABTS radical scavenging activity (ABTS, mg TE/100 g) of free phenolics from rice bran (a) and rice husk (c) fractions and ferric reducing antioxidant power (FRAP, mg TE/100 g) of free phenolics from rice bran (b) and rice husk (d) fractions. Figure S2. Effect of NaOH concentration, time and temperature hydrolysis on ABTS radical scavenging activity (ABTS, mg TE/100 g) of bound phenolics from rice bran (a) and rice husk (c) fractions and ferric reducing antioxidant power (FRAP, mg TE/100 g) of bound phenolics from rice bran (b) and rice husk (d) fractions.
