# Supplementary material for: Optimizing Extraction Conditions of Free and Bound Phenolic Compounds from Rice By-Products and Their Antioxidant Effects
Source: Foods. 2018 Jun 13;7(6):93. doi: 10.3390/foods7060093 (PMC6024898; doi:10.3390/foods7060093)
Supplement: Supplementary file 1 [file foods-07-00093-s001.pdf]

Supplementary Materials: Supplementary materials are available online.

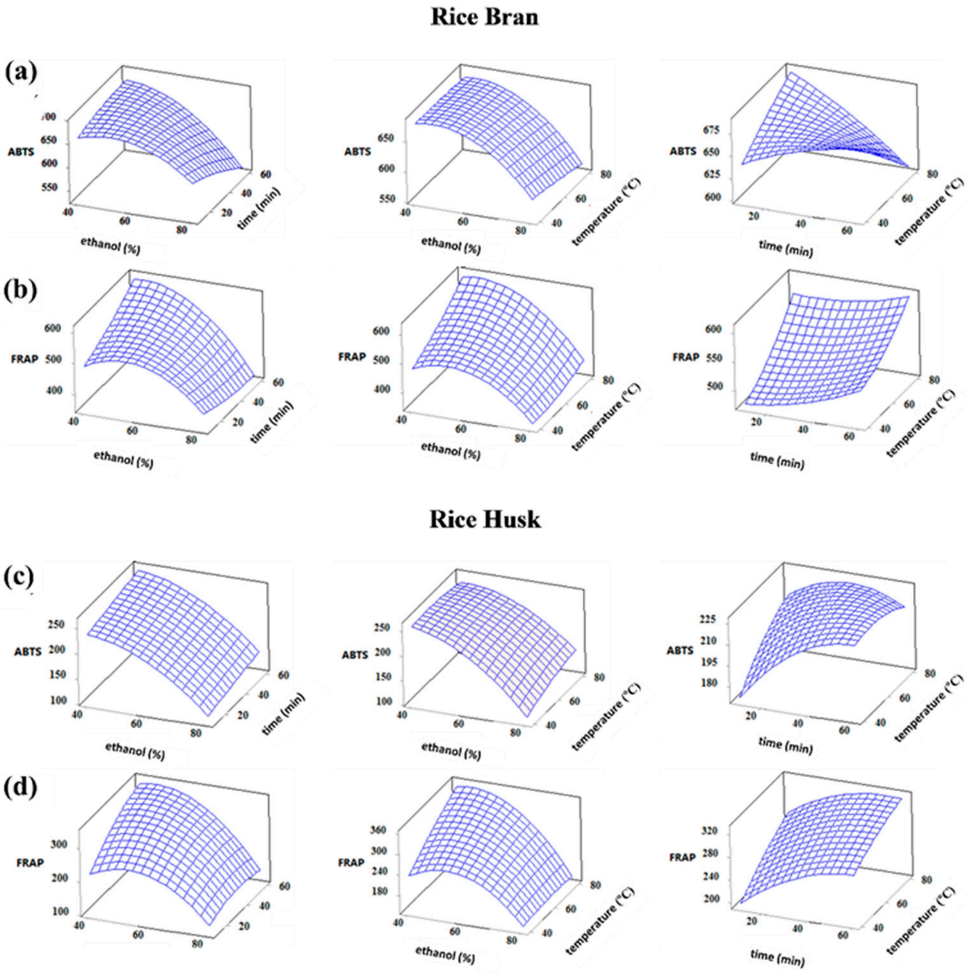

Figure 1s. Effect of ethanol concentration, time and temperature extraction on ABTS radical scavenging activity (ABTS, mg TE/100 g) of free phenolics from rice bran (a) and rice husk (c) fractions and ferric reducing antioxidant power (FRAP, mg TE/100 g) of free phenolics from rice bran (b) and rice husk (d) fractions.

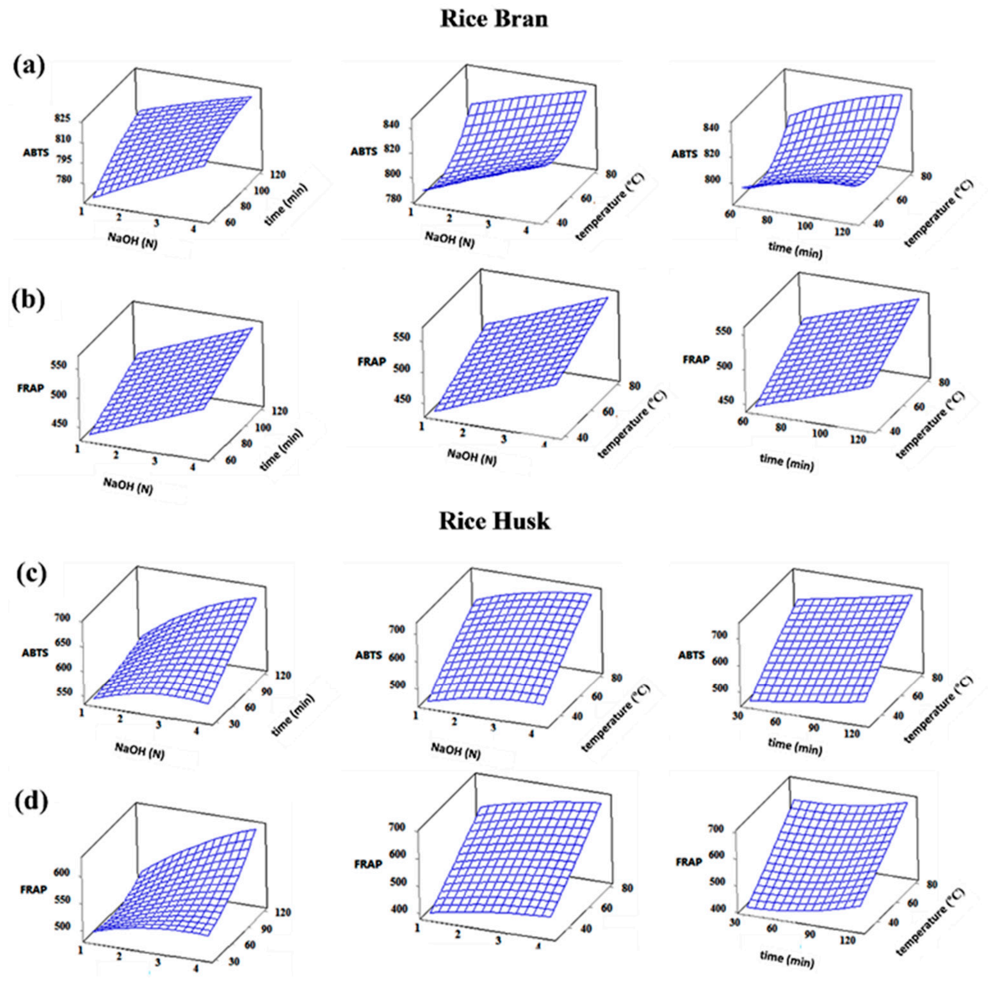

Figure 2s. Effect of NaOH concentration, time and temperature hydrolysis on ABTS radical scavenging activity (ABTS, mg TE/100 g) of bound phenolics from rice bran (a) and rice husk (c) fractions and ferric reducing antioxidant power (FRAP, mg TE/100 g) of bound phenolics from rice bran (b) and rice husk (d) fractions.
